# The Global Landscape of Neuropsychiatric Prescribing Practices of Nurses: A Scoping Review

**DOI:** 10.1111/inm.70101

**Published:** 2025-07-25

**Authors:** M. Naidoo, C. J. Filmalter, W. Cordier

**Affiliations:** ^1^ Department of Pharmacology, School of Medicine, Faculty of Health Science University of Pretoria Hatfield South Africa; ^2^ Department of Nursing Science, School of Healthcare Sciences, Faculty of Health Science University of Pretoria Hatfield South Africa

**Keywords:** anti‐anxiety agents, antidepressant agents, mental disorders, nurse prescribing, nurses, psychopharmacology

## Abstract

Mental healthcare service access in South Africa is currently strained due to, among others, shortages of specialised mental healthcare professionals. The National Strategic Plan for HIV, TB and STIs (2023‐2028) recommends enabling nurses to diagnose, prescribe and dispense neuropsychiatric medication for promoting mental health services. The aim was to explore and describe the existing practices, strengths and challenges for nurses prescribing neuropsychiatric medication globally through a scoping and document review. A standardised search was conducted across PubMed, CINAHL and EBSCOHost electronic databases. An online Google search was conducted across governmental legislative and regulatory websites. The Joanna Briggs Institute scoping review framework was followed using relevant MeSH terms and free‐text words. South African governmental and parastatal documentation relating to relevant regulatory frameworks affecting such prescribing authorisation in South Africa was analysed following Bowen (2009) guidelines. Of 817 citations identified, 20 reports were included. The included reports originated mostly from developed countries, with only one from South Africa. Patients and healthcare professionals were mostly positive towards including the prescription of neuropsychiatric medication in the nursing care model. Prescription of Schedule 5 and 6 controlled substances by nurses is already authorised in the USA and UK. In South Africa, nurses are not yet permitted and will require amendments to the legislative framework that guides nursing practice. Nurses can prescribe neuropsychiatric medications in certain developed countries; however, contextual research is necessary to ascertain whether South African stakeholders will support such an authorisation. Educational and interprofessional concerns will need to be thoroughly assessed to ensure that appropriate competencies are obtained, ensuring the boundaries of the scope of practice. Investigation of potential professional overlap of responsibilities and perceptional biases, as well as transformation of educational platforms, will be needed should such a recommendation come to pass. Furthermore, legislative changes will be required to authorise the prescription of neuropsychiatric medications.

## 1 Introduction

Since 2019, approximately 970 million people worldwide or one in every eight people, are living with a mental health disorder, prominently anxiety and depressive disorders (World Health Organization 2022). Despite being a global burden (Atun et al. 2016), mental health disorders are often neglected within the healthcare system, and as a result are underdiagnosed and undertreated (Kumar et al. 2020). The psychosocial consequences of the COVID‐19 pandemic such as symptoms of anxiety, depression, post‐traumatic stress disorder (PTSD), and sleep disturbances were found to persist for months after infection, indicating the psychological impact of the pandemic despite being over (Zewudie et al. 2021). Since 2022, approximately 25.7% of South Africans have been living with probable depression (Fentahun et al. 2023). Mental health disorders frequently co‐occur with infectious diseases such as human immunodeficiency virus/acquired immunodeficiency syndrome (HIV/AIDS), tuberculosis (TB), and other chronic diseases (Craig et al. 2022). HIV/AIDS and mental health disorders are highly stigmatised, which leads to unnecessary psychological distress and additional trauma to patients (Meyer et al. 2019). A 2021 South African systematic review found that the HIV stigma leads to a positive correlation between depressive symptoms and people living with HIV/AIDS (Powell 2023). Stigmatisation of people living with mental health disorders includes frequent victimisation for their illnesses, mistreatment by society, and unfair discrimination (MacLean and Wetherall 2021). Stigma associated with mental health disorders is a common obstacle to healthcare access and service delivery, thus prolonging the recovery time of the patient (Sartorius et al. 2015).

The treatment available for common mental health disorders includes psychotherapy such as cognitive‐behavioural therapies and pharmacotherapy such as antidepressants and anxiolytics (Moitra et al. 2023; Sheikhan 2023). Despite effective prevention and treatment options present, the increasing prevalence of people living with mental health disorders requires an increase in mental healthcare services to support patients' mental healthcare needs (Hyman et al. 2006). Yet, with a treatment gap for mental health disorders of 92% (Cuijpers et al. 2021), an estimated 75% of South Africans living with common mental health disorders do not receive any form of treatment (Naylor et al. 2012). South Africa is currently experiencing a critical shortage of neuropsychiatric prescribers with an average of 0.31 public sector psychiatrists per 100 000 uninsured population (Cuijpers et al. 2021). There is, however, a higher coverage of nurses compared to psychiatrists, with 80 professional nurses per 100 000 people and 27.2 specialist nurses per 100 000 people, though not all are psychiatric nurses (Cuijpers et al. 2021). Therefore, integrating mental health care within broader health strategies can have wider implications for mental healthcare. In order to address the mental health care needs of patients, standardised screening for anxiety, depression and abuse of drugs and alcohol should be implemented in primary care settings along with expanding nurses' scope of practice to prescribing and dispensing of neuropsychiatric medications (Sorsdahl et al. 2023).

The National Department of Health (NDoH) and the South African National AIDS Council (SANAC) suggested an approach under Objective 1.7 of the National Strategic Plan for HIV, TB and STIs (2023‐2028) to support the urgent need to further mental health‐accredited training of healthcare practitioners (Sorsdahl et al. 2023). The Objective aims to increase clinical responsibilities with regard to service delivery to mental health patients (Sorsdahl et al. 2023). Objective 1.7 is directed to specifically permit trained specialist registered nurses to prescribe and dispense neuropsychiatric medications alongside standardising and executing screening tools which may be used for anxiety, depression and harmful drug and alcohol abuse in primary care facilities (Sorsdahl et al. 2023). In doing so, more practitioners can make a diagnosis, prescribe appropriate medication and treat the mental disorder. In terms of pharmacotherapy, this could increase access to neuropsychiatric medication for patients living with a mental health disorder (Sorsdahl et al. 2023).

Nurse prescribing—not specific to neuropsychiatric prescribing alone—is legally recognised in the United States, United Kingdom, Sweden, South Africa, Australia, Canada, New Zealand, Brazil, France, Ireland, Lesotho, Botswana and Argentina (Docrat et al. 2019). The onset of nurse prescribing is said to offer many benefits for patients and to facilitate and extend service development (South African National AIDS Council 2023)‐2028. However, it is unclear what is currently known about the broader attitudes, perceptions, benefits and detriments to implementation of such an extension of scope of practice.

As the implementation of nurse neuropsychiatric prescribing in the South African context is contentious, the study aimed to explore related literature to determine the perceptions and potential impact of such a practice. Furthermore, to contextualise the potential regulatory considerations, South African documents that guide non‐medical prescription practice in South Africa were analysed.

## 2 Methods

### 2.1 Scoping Review

The scoping review followed the Joanna Briggs Institute (JBI) 2020 method (Ayuso 2018) which ensured a structured search of databases and a thorough exploration of the literature that related to nurses prescribing neuropsychiatric medication.

### 2.2 Guiding Question and Search Strategy

The guiding research question for the scoping review was ‘What is known about existing practices of nurses prescribing neuropsychiatric medication to patients living with a mental health disorder?’. The researcher conducted a literature search to identify relevant citations using free‐text keywords and Medical Subject Heading (MeSH) terms in the following electronic databases: PubMed, CINAHL and EBSCOHost. The Population, Concept, Context (PCC) framework was used to determine relevant terms alongside Boolean operators (Table 1) and refined with the assistance of an information technologist from the Library Services of the University of Pretoria. All formats of primary studies were included in the study if they adhered to the following inclusion criteria: (i) focused on the nursing profession, (ii) prescribing practices by nurses, (iii) mental health disorders and (iv) full‐text, English articles. No date range limit was applied. The following exclusion criteria was applied: (i) literature that was not in English, (ii) not full‐text, (iii) review articles, (iv) articles outside of the nursing profession or neuropsychiatric domain focusing on antipsychotics. The researchers are proficient in English, therefore focused on including original studies relevant to the research question. The exclusion criteria allowed for a comprehensive assessment of primary research and maintained consistency in data extraction and analysis. As the Strategic Action Plan does not speak towards antipsychotic prescription as part of the recommendation, literature with a sole focus on antipsychotics were also excluded.

**TABLE 1:** Search terms according to population, concept, context (PCC) framework.

	PCC description	MeSH term
Population	Nurses	‘nurse practitioners’, ‘nursing staff’, ‘nurse's role’, ‘psychiatric nursing’, ‘non‐medical prescribing’, ‘attitude of health personnel’, ‘knowledge’, ‘practice patterns, nurses’
Concept	Neuropsychiatric prescribing	‘antidepressive agents’, ‘antipsychotic agents’, ‘anti‐anxiety agents’
Context	Mental health	‘mental health’, ‘mental disorders’, ‘depression’, ‘anxiety disorders’, ‘bipolar disorder’

### 2.3 Screening Approach

The scoping review followed a two‐step blinded screening process which included a title and abstract assessment, followed by a full‐text examination of relevant articles. All citations were imported into Rayyan.ai to facilitate reviewer‐blinded screening, after which duplicate removal was applied. A three‐reviewer approach (MN, WC, Dr. Champion Nyoni) was used to screen titles and abstracts against the eligibility criteria for inclusion, exclusion (with reasons) or further discussion. After citation screening, the decision was unblinded for consensus discussion. All included citations, after agreement was reached, were sourced as full‐text articles and uploaded into Rayyan.ai for full‐text review. During full‐text blinded‐reviewing, two reviewers (MN, WC) read the papers in full to determine their eligibility. After the review was completed, consensus discussion was held until agreement was reached between the reviewers. The agreement of the screening and full‐text review was determined using the Statistical Package for the Social Sciences (SPSS) software using a Fleiss‐kappa and Cohen‐kappa agreement statistics, respectively. The reporting of the review followed the Preferred Reporting Items for Systematic Reviews and Meta‐Analyses extension for Scoping Reviews (PRISMA‐ScR) flow diagram.

### 2.4 Data Extraction and Analysis

Data charting was done by each reviewer (MN, WC) to extract information from all included articles using a Google form. The following fields were captured: Author(s), Level of evidence, Article title, Aim, Study design, Population and Study findings. Analysis of the scoping review was done using descriptive quantitative and qualitative means as appropriate for the findings of the study. Quantitative information obtained from the studies were described as means, standard deviations and frequencies. Qualitative analysis was used where appropriate to thematically describe the findings of the studies facilitated by iterative reading. The levels of evidence of each publication were extracted based on the research design, quality of the study, and applicability to patient care following Melnyk and Fineout‐Overholt (2023) seven levels of evidence hierarchy, where a lower level of evidence has a lower risk of bias (Peters et al. 2022).

### 2.5 Document Analysis

A document analysis following the six‐step framework of Bowen (2009) (Melnyk and Fineout‐Overholt 2023) was conducted to determine the regulatory frameworks and platforms that govern nurse prescription practice in South Africa. The document analysis enhanced the scoping review by analysing the legislative regulations/guidelines that govern non‐medical nurse prescription should neuropsychiatric prescribing by nurses be authorised in South Africa. The documents selected were those of South African relevance to prescribing of medications and the scope of practice of nurses. These regulations were investigated by sourcing gazettes and guiding documents via an online Google search from governmental (NDoH) and parastatal (South African Pharmacy Council [SAPC], South African Health Products Regulatory Authority [SAHPRA] and South African Nursing Council [SANC]) websites. Where reference was made within these documents to another act, indirect sourcing was done where relevant. A three‐step process was followed where the documents were read for familiarisation, thorough reading, and lastly interpretation (Melnyk and Fineout‐Overholt 2023). Relevant information from the evaluated documents was extracted based on the sections of the acts or guidelines that govern the regulations as direct, literatim quotations. Coding was informed by the scope of practice of the nurse in relation to prescribing and the authority of the prescribing regulations. ATLAS.ti Scientific Software Development GmbH version 8.6.22002–2024 was used for coding and quoting the sourced documents.

### 2.6 Ethics Approval

The following study obtained ethics approval from the Research Ethics Committee (REC) at the University of Pretoria, School of Medicine, Faculty of Health Science. The conduction of the two‐process screening method was blinded until the reviewers completed screening to allow for an unbiased decision when selecting citations. During the conduction of the document analysis, direct quotations obtained from the documents were discussed contextually using coding to establish themes. Verbatim quotation of the acts and guidelines was done to ensure transparency and reliability, where the multiple reviewer approach allows for inter‐rater discussion of the information.

## 3 Results

### 3.1 Search Results and Publication Characteristics

**FIGURE 1:**
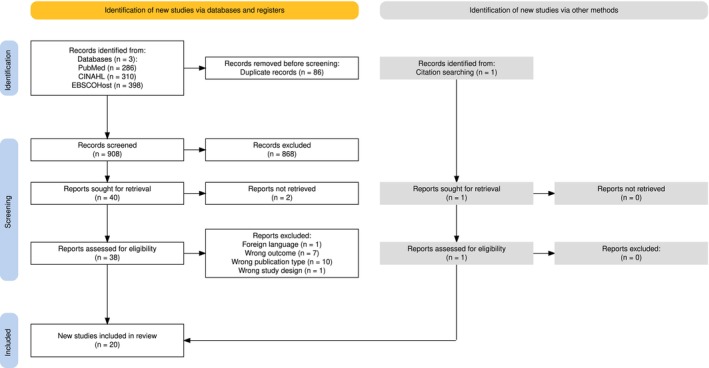
Preferred reporting items for systematic reviews and meta‐analyses extension for Scoping Reviews (PRISMA‐ScR) flow diagram used for data reporting (Haddaway et al. 2022).

The literature search yielded 817 unique citations, out of which 20 publications were eligible for inclusion. Details of the screening process are summarised in the PRISMA‐ScR diagram (Figure 1). The Fleiss‐kappa statistic measuring the agreement between the three reviewers (MN, WC, Champion Nyoni) for the abstract and title screening was calculated to be 0.442, indicating moderate agreement (Bowen 2009). The Cohen‐kappa statistic measuring the agreement between the two reviewers (MN, WC) for the full‐text screening was calculated to be 0.075, indicating slight agreement (Bowen 2009).

The mixed methods appraisal tool (MMAT) version 2018 was used to appraise the quality of all included articles to ensure that the findings obtained from the included articles are clearly evaluated and thematically synthesised (Melnyk and Fineout‐Overholt 2023). According to the MMAT quality appraisal (Table 2), the majority of studies were considered of good quality given their adherence to the set criteria. However, approximately seven studies had notable limitations, particularly in their sampling strategy, representativeness of a population, and response bias; which was also acknowledged within the publications' limitations. For instance, Flenniken (1997), Talley and Richens (2001), Shell (2001), Wolfe et al. (2008), Garrison and Levin (2000), and Gumber et al. (2011) had clear methodological gaps (Alexander and Schnell 2019; Chai et al. 2024; Fisher and Vaughan‐Cole 2003; Flenniken 1997; Garrison and Levin 2000; Gumber et al. 2011). For example, Flenniken (1997) surveyed only 42 NPs across various settings without a defined sampling strategy (Alexander and Schnell 2019), and Talley and Richens (2001) used a sample size of *n* = 88 (across 7 states) yet, did not clearly include the recruitment process of participants (Chai et al. 2024). Furthermore, Shell (2001), Wolfe et al. (2008), Garrison and Levin (2000) and Gumber et al. (2011) showed either a high non‐response rate of participants or were unclear about the specific sampling processes (Fisher and Vaughan‐Cole 2003; Flenniken 1997; Garrison and Levin 2000; Gumber et al. 2011). Lastly, the single quantitative randomised clinical trial did not use true random assignment or blinded assessors which could increase the risk of potential bias (Jacobs 2005). Therefore, the abovementioned studies' findings were required to be interpreted with caution as the samples may not be representative of an entire population, specifically the NP population, and due to the limited sampling, NP prescribing patterns and trends could be over‐ or under‐estimated (Alexander and Schnell 2019; Chai et al. 2024; Fisher and Vaughan‐Cole 2003; Flenniken 1997; Garrison and Levin 2000; Gumber et al. 2011; Jacobs 2005).

**TABLE 2:** Quality appraisal using MMAT version 2018. An X represents adherence to the checklist parameter, while an absence thereof equates to a lack of adherence. Can not tell signifies too little information or ambiguity to explicitly decide.

Quantitative descriptive							
	Sampling relevant	Representative sample	Appropriate measurements	Low nonresponse bias	Appropriate statistical analysis	Clear research questions	Data collection addressed research questions
Flenniken (1997)	Can't tell		X		X	X	X
Garrison and Levin (2000)	X	Can't tell	X	Can't tell	X	X	X
Shell (2001)	X	Can't tell	X		X	X	X
Talley and Richens (2001)	X	Can't tell	X		X	X	X
Wolfe et al. (2008)	X	Can't tell	X		X	X	X
Gumber et al. (2011)	X	Can't tell	X	Can't tell	X	X	X
Poot et al. (2020)	X	X	X	X	X	X	X
Chai et al. (2024)	X	X	X	X	X	X	X

Quantitative non‐randomised							
	Representative participants	Appropriate measurements	Complete outcome data	Confounders accounted for	Exposure as intended	Clear research questions	Data collection addressed research questions
Yang et al. (2018)	X	X	X	X	X	X	X
Alexander and Schnell (2019)	X	X	X	X	X	X	X
Yang et al. (2021)	X	X	X	X	X	X	X

Quantitative randomised controlled trial							
	Randomization appropriate	Comparable at baseline	Complete outcome data	Assessors blinded	Adherence to intervention	Clear research questions	Data collection addressed research questions
Norman et al. (2010)		Can't tell	X		X	X	X

Qualitative							
	Appropriate approach	Adequate data collection methods	Findings adequately derived from data	Interpretation of results sufficient	Coherence between qualitative data sources, collection, analysis and interpretation	Clear research questions	Data collection addressed research questions
Allsop et al. (2005)	X	X	X	X	X	X	X
Jones (2005)						X	
Jones et al. (2007)	X	X	X	X	X	X	X
Ross (2015)	X	X	X	X	X	X	X
Jacobs and Mkhize (2021)	X	X	X	X	X	X	X

Mixed‐methods							
	Adequate rationale	Different components effectively integrated	Qualitative and quantitative components adequately interpreted	Divergences and inconsistencies adequately addressed?	Adherence to the quality criteria	Clear research questions	Data collection addressed research questions
Fisher and Vaughan‐Cole (2003)	X	X	X	Can't tell	X	X	X
Jacobs (2005)	X	X	X	Can't tell	X	X	X
Snowden (2010)	X	X	X	Can't tell	X	X	X

Most of the studies were quantitative (*n* = 12), with fewer qualitative (*n* = 5) and mixed method (*n* = 3) study designs. Most literature originated from the USA (*n* = 11) and UK (*n* = 7), with a single publication each from South Africa and New Zealand. Based on the gathered literature within the scholarly base, publications began from as early as 1997 up until 2024, however, were sporadically published with no particular trend observed. The level of evidence of studies included level 2 (*n* = 1), level 3 (*n* = 2), level 4 (*n* = 3) and level 6 (*n* = 13). The summarised main findings from the data extraction process are presented in Table 4.

### 3.2 Primary Themes Identified From Literature

Six main themes were identified between multiple publications: (i) prescribing patterns (*n* = 14), (ii) perceptions or attitudes (*n* = 9), (iii) auditing (*n* = 4), (iv) further education (*n* = 9), (v) benefits (*n* = 6) and (vi) barriers (*n* = 4). The themes are further defined in Table 3.

**TABLE 3:** Definitions of identified themes.

Theme	Definition
Prescribing patterns	The extent and profile of medication prescribed by healthcare professionals, trends in type and quality of drugs, and compliance with standard treatment guidelines
Perceptions or attitudes	The views and opinions of healthcare professionals and patients towards nurse prescribing
Auditing	Laws and regulations that are required to be implemented to authorise neuropsychiatric prescribing by nurses
Further education	Required education and training to improve nurses' knowledge to ensure safe neuropsychiatric prescribing practices
Benefits	The advantages and positive outcomes as a result of authorising neuropsychiatric prescribing by nurses
Barriers	The disadvantages and negative outcomes as a result of authorising neuropsychiatric prescribing by nurses

**TABLE 4:** Summary of all included publications.

Author(s)	Level of evidence^a^	Article title	Aim	Study design	Population	Study findings
Flenniken (1997)	6	Psychotropic prescriptive patterns among nurse practitioners in nonpsychiatric settings	Exploration of mental healthcare treatment among primary care nurse practitioners	Quantitative design	Purposive sampling: 42 out of 60 nurse practitioners were drawn from a published advanced practice nurse directory (certified nurse practitioner; direct care provider; prescriptive privileges; nonpsychiatric settings)	Psychiatric disorders encountered: 95% Patients with mainly depression and anxiety. Referrals: 93% Patients to mental health specialists. Prescribed psychotropic medications: Antidepressants (59.5%), anxiolytics (59.5%), sedative/hypnotics (42.8%), Ritalin (30.9%), antipsychotics (23.8%), and antimanic drugs (7.1%). Prescribing comfort: 25% ‘very comfortable’, 60% ‘fairly comfortable’ and 5% ‘somewhat uncomfortable’. Psychopharmacology continuing education received: 50% Received ≤ 3 h/year. Psychopharmacology continuing education required: 68% Required, 13% were uncertain and 19% did not feel the need.
Garrison and Levin (2000)	6	Factors affecting prescribing of the newer antidepressants	To determine whether differences exist in prescribing patterns for the newer antidepressants by surveying various prescriber types and specialties	Quantitative design	Purposive sampling from New York State Coalition of Nurse Practitioners; Mixed prescriber population, of which 102 (26%) were nurse practitioners and others were physician assistants and physicians in family medicine, primary care, general practice, and internal medicine; and psychiatrists	Factors affecting prescribing selection: Adverse effects are a moderate priority (50%). Prescribing scenarios: (50% or 6/12) differences in scenarios for third‐generation antidepressant (TGA) selection. Prescriber statements: Nurse practitioners chose familiar TGAs (7/10) with differences among groups statistically significant (*p* = 0.0009), not as an arbitrary selection (1/10), not according to patient request (4/10), have professional interaction with pharmaceutical representatives that provide TGA information (5/10), and based on labelled indications (7/10) with differences among groups statistically significant (*p* = 0.002). Frequency: Nurse practitioners (NPs) often prescribe < 1 (23%) or 1‐5 (60%) TGAs more frequently than other prescriber groups. Practice sites: Generally, in less dense areas (practice site population < 50 000 [52%] and 50 000‐250 000 [32%])
Shell (2001)	6	Antidepressant prescribing practices of nurse practitioners	To determine factors that nurse practitioners consider when choosing antidepressants, the frequency of prescription, the types of antidepressants, and the health problems treated with antidepressants. Furthermore, perceived competence was determined in choosing appropriate antidepressants and the perceived need for further education about antidepressants	Quantitative design	Simple randomised sampling via number‐generated randomization of mailing list; 44 nurse practitioners (36 family nurse practitioners, 4 paediatric nurse practitioners, and 4 undefined nurse practitioners) with direct patient care of at least 30 h per week and authority to prescribe	Mental health disorders: 29% Patients recorded. Antidepressant prescriptions written per week: NPs wrote between 1 and 100 with an average duration of 6 to 52 weeks. Informed about choosing antidepressants: 71% ‘sufficiently informed’ and 56% ‘required further education about antidepressants’. Prescribed antidepressants: Fluoxetine (43%), paroxetine (39%), amitriptyline (34%) and sertraline (25%). Commonly presented conditions: Depression (91%), and social phobia/panic (45%). Factors affecting antidepressant choice: Cost, drug interactions, medical conditions, adverse effects, lethality in overdose, patient experience, age, knowledge of antidepressants, sample availability, dosing frequency and efficacy.
Talley and Richens (2001)	6	Prescribing practices of advanced practice psychiatric nurses: Part I‐Demographic, educational, and practice characteristics	To identify and describe prescribing practices of advanced practice psychiatric nurses	Quantitative design	Convenience sampling; 150 of 290 nurse respondents (88 prescribers; 62 nonprescribers)	Demographics: (88.5%) Female, (98.9%) Caucasian, (81.6%) with Masters qualifications, (25.3%) certified in specialties. Practice: Community mental health center (38.6%), independent practice (23.9%) and hospital/inpatient settings (12.5%). Physician collaboration was required (18.4%), while (81%) had a voluntary consultation relationship with a physician collaborator. Restrictions: Specialty practice‐scope of practice (57%), statute formulary (42%), no controlled substances (26.1%) and formulary and protocol (10.2%). Controlled substance type allowed: Two‐to‐five (14.8%), three‐to‐five (30.7%), four and five (2.3%), and only five (18.2%) of controlled substances. Services: Referred to client's NP or medical doctor (61.4%), requested laboratory services directly (86.3%), psychiatric admission through emergency departments (33.7%). Commonly prescribed medication: SSRIs (2.59/3), TCAs (2.50/3), antiparkinsonian (2.20/3), lithium (2.18/3) and phenothiazine (2.02/3). Side effects: Anticholinergic (2.59/3), weight gain (2.24/3), extrapyramidal (2.09/3), orthostatic hypotension (1.82/3), and sexual difficulties in terms of frequency (1.78/3). Reasons for medication choice: Previous side effects (4.74/5); previous response to medication (4.57/5); current medical problems (4.61/5); suicidality and addiction (4.35/5). Drug information sources: Clinical experience (4.50/5), continuing education (4.46/5), drug textbooks (4.15/5), conference and meetings (4.20/5); psychiatrists (3.76/5). Perceptions: 9% (out of 46 participants) Worked in areas prohibiting prescribing. (39.1% always; 34.8% most often) prescribed generic drugs, and 13% experienced difficulties with prescription filling due to: (1/6) drug interaction identified; (2/6) pharmacist didn't honour prescription; (3/6) unspecified reasons. Comparison of nurse to physician practice: (54.5%) nurses regarded their practice as like physicians, (43.3%) were more cautious, (17.4%) believed they prescribed the same number of medications, and (82.6%) believed they prescribed less.
Fisher and Vaughan‐Cole (2003)	3	Similarities and difference in clients treated and in medications prescribed by APRNs and psychiatrists in a CMHC	To compare the prescribing practices of psychiatrists and advanced practice registered nurses (APRNs) by analysing the types and numbers of medications prescribed, as well as the similarities and differences in the clients treated by each professional group in a community mental health center (CMHC)	Mixed method design	Purposive sampling: 5507 adult clients who had medication evaluations and/or medication management contacts with APRNs or psychiatrists in the year 2000. Simple randomised subsample of 120 randomly selected charts of adult outpatient clients (60 for APRNs and 60 for psychiatrists)	Patient severity: Similar ratings between psychiatrists and APRNs, however, greater severity for those treated with both. Diagnoses: APRNs treated more clients with drug/alcohol abuse problems, and psychiatrists treated more childhood conditions, particularly attention deficit hyperactivity disorder (ADHD). Prescriptions: APRNs prescribed more selective serotonin reuptake inhibitors (SSRIs) compared to psychiatrists (65% vs. 38%). Psychiatrists prescribed more tricyclic antidepressants compared to APRNs (19% vs. 4%). Duration of visit: Significant difference (*p* = 0.04) in the median visit duration between patients seen by an APRN and psychiatrist respectively (30 min vs. 20 min).
Allsop et al. (2005)	6	Supplementary prescribing in mental health and learning disabilities	To describe the experiences of nurses working in mental health and learning disabilities who formed part of the first cohort in the United Kingdom to undertake the supplementary nurse prescribing course	Qualitative design	Nine nurses (three learning disability nurses; five mental health nurses; one paediatric nurse) from the South Staffordshire NHS trust	Support and concern: Nurses needed support to gain supplementary prescribing rights, but concerns were expressed of regulations on clinical practice as prescribers. Initial fear: Fear acknowledged during their first prescription in relation to adverse effects and the responsibility. Education: Acknowledgement of the importance of sufficient education needed for prescribing. Patient acceptance: Patients accepted nurse prescribers and expressed increased and easier access to service delivery.
Jacobs (2005)	3	Treatment of depressive disorders in split versus integrated therapy and comparison of prescriptive practices of psychiatrists and advanced practice registered nurses	To determine differences in adherence to medication for the treatment of depressive mood disorders whether the patient was involved in split or integrated therapy, and if the patient was treated by a psychiatrist or an advanced practice registered nurse in psychiatry with prescriptive authority	Mixed method design	Convenience sampling: 122 adults (age range, 20–60 years) with a depressive disorder and who were voluntarily being treated by either a psychiatrist (6) or a psychiatric nurse (6) with prescriptive authority	Medication adherence: No difference between split^b^ vs. integrated^c^ therapy, or between psychiatrists and APRNs. Patients seen: Nurses saw more in integrated therapy, while psychiatrists saw twice as many in split therapy. Similar SSRI prescribing rate: psychiatrists (split 68%; 75% integrated), nurses (split 75%; 73% integrated). Similar secondary antidepressant prescription: Split therapy (39% vs. 31%), while psychiatrists prescribed more in integrated therapy (55% vs. 32%). Combinational therapy use: Psychiatrists (40% in integrated therapy), and nurses (48% use one medication). Prescription of benzodiazepine and mood stabilisers: Split therapy: Nurses (20%; 20%) and psychiatrists (15%; 35%) in split therapy. Integrated therapy: Psychiatrists (55% vs. 27%; 45% vs. 22%).
A. Jones (2005)	6	Supplementary prescribing: Potential ways to reform hospital psychiatric care	To explore perceptions of nurses and psychiatrists towards the potential application of supplementary prescribing at acute psychiatric wards	Qualitative design	Purposive sampling: Nurses (19) and psychiatrists (7) who worked at a district general hospital in North Wales	Nurse perception of supplementary prescribing: Increased service delivery, quality of service and pharmacotherapy access. Nurse empowerment: Nurses could make treatment changes, involve patients in decision‐making of treatment, facilitate one‐to‐one treatment with a nurse prescriber, understand the unique individual treatment better, promote better patient relationships, allow for earlier discharge, and improved recovery. Patient perception: Some patients may be nervous or unclear of nurse prescribers. Psychiatrists' perception: Nurses lack diagnostic skills, require support from the psychiatrist which would make nurses' schedules more flexible and unstructured. Nurse eligibility: Disagreement of which category of nurses should be allowed to supplementary prescribe (more experienced, higher categories, or those showing aptitude). Nurses should receive proper training, understand their role and have leadership/communication skills. Implementation challenges: Mixed opinions of how supplementary prescribing could increase workload or necessitate reshuffling to enable proper prescribing. Responsibility concerns: Altered dynamics between nurses, create reporting lines or disconnect with general nursing duties. Prescribing concerns: Role and expertise of the nurse, medication errors, and potential litigation though psychiatrists believed this would not impact service delivery.
Jones et al. (2007)	6	Mental health nurse supplementary prescribing: Experiences of mental health nurses, psychiatrists and patients	To explore the views of patients, mental health nurses and psychiatrists involved in mental health nurse supplementary prescribing	Qualitative design	Purposive sampling: Psychiatrists (11), mental health nurses with prescribing authority (11) and patients who have been prescribed medication by a nurse (11)	Perception to nurse prescribing: 33 out of 34 (97%) participants responded positively. Demographics: Patients, nurses and psychiatrists had mean ages 43, 44 and 47 years respectively. Male: (55%) patients and (58%) psychiatrists. Female: (63%) nurses. Patients were receiving mental health treatment for a mean of 11 years. Experience: Both mental healthcare nurses and psychiatrists had means of 21 and 18 years of clinical experience. Little psychopharmacology education received during training. Prescribing patterns: Nurses prescribed for a mean of 2 patients over a period of 4 months with more than 80 written prescriptions (psychotropic medications, including anti psychotics and antidepressants). Perceptions: All healthcare providers acknowledged mistakes, while psychiatrists believed that nurses lacked experience. No healthcare provider disagreed with nurse prescribing but believed that continuing education was needed, and the role should be redesigned for the service. Barriers: Risk of nurses making mistakes, although no prescribing mistakes were detected. Supervision: Participants indicated clinical supervision was needed for professional development, audit and service evaluation. Person‐centered approach: Suggested as a professional framework for nurse prescribers of psychiatric medication.
Wolfe et al. (2008)	6	Psychopharmacologic first‐line strategies in the treatment of major depression and psychosis: A survey of advanced practice nurses	To examine the prescribing practices of advanced practice nurses in a psychopharmacology continuing education program	Quantitative design	Purposive sampling: 183 of 247 nurse respondents	Demographics: Most prescribing advanced practice nurses were psychiatric‐mental health specialists. Most had a Masters degree and worked in outpatient facilities or private practice (similar between prescribers and non‐prescribers). Commonly treated condition: 95% provided direct patient care. Depressive disorders were the most common, followed by mood disorders, anxiety and others. Medication management: (65.9%) prescribed psychotropic medications, for depression (97.4%), mood disorders (84.2%), schizophrenia and psychotic disorders (71.1%), anxiety (89.4%) and PTSD(73.5%). (88.1%) had patients that experienced side effects. SSRI as first‐line treatment: (97.3%) prescribed SSRI for major depression. (61%) increased the dose of the SSRI if ineffective. (17%) switched to another SSRI or (11%) to a non‐SSRI. (80%) added trazodone‐like drugs if insomnia was experienced as side effect.
Norman et al. (2010)	2	A comparison of the clinical effectiveness and costs of mental health nurse supplementary prescribing and independent medical prescribing: a post‐test control group study	To compare the treatment costs, clinical outcomes and satisfaction of patients in receipt of mental health nurse supplementary prescribing in receipt of independent prescribing	Quantitative design	Purposive sampling: Matched samples of 90 patients with a primary diagnosis of depression, anxiety, or schizophrenia from five mental health NHS trusts treated either with nurses or medical practitioners (45 patients per group)	Patient demographics: No significant differences in patient age or chronicity between groups, nor SIMS, MARS, WSAS, Clinical Global Impression Scale, BDI‐II, one item depression scale^d^, CSQ‐8, and side effects. Patient satisfaction: Significantly higher satisfaction with the nurse prescriber (3.42 vs. 3.04)^e^ with 95% confidence interval of mean difference (0.03 to 0.72). Service costs: The service use costs per patient were £803 higher for the nurse prescribers. This included total cost with and without informal care per patient. Frequency of contact: Mean frequency of contact with other therapists were higher in medical practitioners (35 vs. 10.1)^e^ compared to nurses.
Snowden (2010)	6	Integrating medicines management into mental health nursing in UK	To analyse the process of developing competence in mental health nurse prescribing to generate an evidence‐based framework for delivery of safe and appropriate medicine management	Mixed method design	Purposive sampling: 122 mental health nurses attended the two conference presentations of which 31 were practicing nurse prescribers	Nurse demographics: Among 31 nurse prescribers, most were band 6 (low order; 14) or band 7 (higher order; 12) and mainly worked as community mental health nurses or community psychiatric nurses were also prevalent. Knowledge and experience of prescribers: Themes were broadly coherent with experiences of mental health nurse prescribers and may also have wider applications. Nurses' understanding of medicines improved after they took the prescribing course. Perceptions: Mixed views about stopping or initiating medications. Nurses feared whether doctors would support discontinuation of medication, or addition of medication would be best. Opinions arose about prescribing being a function of the role than it being the intent. Furthermore, the patient's best interest was more important. Model of competence of nurse prescribing: The model was positively received, with emphasis placed on the understanding needed, and that there should be an established therapeutic relationship that allows for integration of therapies.
Gumber et al. (2011)	6	Non‐medical prescribing: Audit, practice and views	To review compliance with existing UK Standards of non‐medical prescribing practice in a UK Mental Health Trust, and determine the views of local non‐medical prescribers engaged in prescribing about the Trusts existing monitoring arrangements of the UK Standards	Quantitative design	Purposive sampling; Nurses (18) and pharmacists (2) from the Trust register	Medications prescribed: Antipsychotics, hypnotics, anxiolytics, mood stabilisers, antidepressants (mostly SSRIs), stimulants, opiate dependency treatments, and antidementia medication were prescribed at variable frequencies. Compliance: Most non‐medical prescribers were compliant with the UK standards (ranging from 75 to 100% depending on which item was considered). Perceptions towards non‐medical prescribing: Non‐medical prescribers expressed confidence in prescribing independently. Prescribing was evidence‐based, person‐centered and improved the nurse–patient relationship, but uncertainty about whether it promoted physical health. Believed to have increased workload. Training and supervision: All to most agreed that they had sufficient knowledge of side effects, dosage, drug interactions, withdrawal effects, and physical health monitoring. They could seek advice from psychiatrists where needed. Mistakes were rarely made, but there were shortfalls in training and supervision. Interprofessional relationships: Agreement (45%), disagreement (20%) and strong disagreement (30%) with non‐medical prescribers having conflicts with psychiatrists, and disagreement that prescribing should be done by medical doctors when possible (55%). Benefits: Non‐medical prescribing increased access to medication and benefited mental healthcare, but there were also mixed fears about litigation.
Ross (2015)	6	Mental health nurse prescribing: The emerging impact	To identify the impact of nurse prescribing on clients in mental health settings and to identify emerging themes	Qualitative design	Convenience sampling: Mental health nurse prescribers (35), pharmacist prescribers (3), nurse managers (2), consultant psychiatrists (7), general practitioner (1) and clients (9) which included 13 interviews and nine focus groups	Nurse prescriber‐patient relationship: Relationships with nurse prescribers had changed, with more trust and ease for patients, so ultimately beneficial. Most agreed that patients may want to see the nurse prescriber rather than alternative medical practitioners or doctors as the relationship was important. Concordance: Nurses discussed the treatments with patients, and believed they brought them into the discussion of the pharmacotherapy. Psychiatrists agreed that they lacked concordance with their clients, and that nurses might achieve it better. Nurses were also better (from all groups) at discussing information with patients in a language and at a level they understand, can dedicate more time to them, provide better information about side effects and the management thereof, and generally led to better perceived adherence. Power: Nurse prescribers felt more empowered and confident with their skills and believed that it also empowered their patients due to bringing them into the treatment discussion. Treatment approach: Nurse prescribers believed their model of caring had not been altered from curing, which a psychiatrist also agreed with. They also believed they were unlikely to prescribe more, with pharmacotherapy being a later or last option. Clients seems to appreciate that non‐pharmacological care was being provided first, and that holistic care was achieved. Unprescribing of medication: Nurses believed that discontinuing prescriptions or reducing medication doses was important, particularly through education. Medication review was considered important, particularly with inappropriate prescribing and polypharmacy.
Yang et al. (2018)	6	Comparing nurse practitioner and physician prescribing of psychotropic medications for Medicaid‐insured youths	To describe psychotropic medication prescribing practices of nurse practitioners and physicians for Medicaid‐insured youths in 2012‐2014 in a mid‐Atlantic state where nurse practitioner prescribing is authorised	Quantitative design	Purposive sampling: 1034798 dispensed psychotropic medications prescribed by nurse practitioners and physicians for 61 526 enrolled Medicaid‐insured youths (2—17 years)	Patient demographics: NPs saw more female, white, Medicaid‐eligible by Temporary Assistance for Needy Families (TANF) patients in small metropolitan or non‐metropolitan/rural areas and were generally less in foster care or receiving Supplemental Security Income (SSI). Number of prescriptions of psychotropic medication: Increase in number of dispensing (from 346 922 to 349 080) between 2012 to 2014. Increase in prescriptions by psychiatric NPs (from 5.9% to 8.8%) and by non‐psychiatric NPs (from 4.9% to 6.3%). In contrast, decrease in prescriptions by psychiatrists (from 56.9% to 53.0%) and by non‐psychiatric physicians (from 32.3% to 31.8%). Healthcare provider: Youths diagnosed with depression or anxiety were more commonly treated by NP‐only than by physician‐only as indicated in the agent of record (Odds ratio adjusted; AOR = 1.33), whereas youths with two or more psychiatric comorbidities were significantly more commonly treated by both NP and physician providers (AOR = 1.44). Prescribing practices: Psychiatric specialists prescribed the bulk of antidepressants (82.0%) and lithium (92.3%), with much lower prescribing by non‐psychiatric specialists (18.0% and 7.7%, respectively). Antipsychotic orders originated from psychiatric specialists 7.4 times more than from their nonpsychiatric specialty counterparts, whether physician or NP. Specialists were more likely to prescribe antipsychotics, antidepressants and lithium, while non‐specialists prescribed greater proportions of ADHD and anxiolytic/hypnotic medication.
Alexander and Schnell (2019)	6	Just what the nurse practitioner ordered: Independent prescriptive authority and population mental health	To examine whether relaxing occupational licensing to allow nurse practitioners to prescribe medication without physician oversight improves population mental health	Quantitative design	Purposive sampling: Retrospective data from seven sources (U.S. Mortality Files, the Behavioural Risk Factor Surveillance System [BRFSS]), and prescription data from IQVIA's Xponent database in the United States of America on independent prescriptive authority for nurse practitioners with administrative data on mental health‐related mortality, and detailed data on prescriptions filled at retail pharmacies	Increase in prescriptions among Medicaid beneficiaries in underserved counties: Antipsychotic prescriptions (33%) and antidepressant prescriptions (50%). Impact on mental health and mortality in underserved areas: Self‐reported mental health increased with NP independent prescribing in undeserved states and a decrease of mental health‐related deaths per quarter was seen in underserved counties and low‐education individuals in underserved counties, respectively (5.83 and 3.2) Number of poor mental health days: Reduction was seen for underserved counties (0.29 days; 9% of the mean) and individuals with low education in underserved counties (0.41 days; over 10% relative to the mean), and even for more than 21 days in poor mental health for these two cohorts (0.01 and 0.01 days, respectively). Prescription rates: Increase in antipsychotics and opioids in all payers per capita (0.01 and 0.04), and for Medicaid‐insured individuals for antidepressants and antipsychotics (0.16 and 0.04 per capita, respectively).
Poot et al. (2020)	4	Potentially inappropriate medicine prescribing by nurse practitioners in New Zealand	To describe potentially inappropriate medicines prescribing to older adults by nurse practitioners in New Zealand	Quantitative design	106 NPs that prescribed medicines to 12 410 patients aged > 65 years	Prescription patterns by NPs: There were 106 of 129 NPs that prescribed medicines to 12 410 patients aged > 65 years. One third of the patients were prescribed > 1 PIMs. (68.4%) were prescribed one PIM; (21.9%) two PIMs; (7.1%) three PIMs; and (2.6%) were prescribed > 4 PIMs. Mean prescription of 81.5 medications with average of (14.9%) PIMs prescribed by NPs. Patient groups receiving PIMs: NPs with mental health and addiction scopes had the highest proportion (33.5%), and primary scope of practice (15.4%). Long‐term condition scope was (12.3%) and older adult scope was 11.1% (*p* < 0.001). Commonly prescribed medications: The most common Beers 2015 PIMs prescribed were hypnotics (3.8%), tricyclic TCAs (3.3.%; amitriptyline being common) and benzodiazepines (2.9%), compared to proton pump inhibitors as the highest prescribed potentially inappropriate medication (15.7%)
Jacobs and Mkhize (2021)	6	Experiences of advanced psychiatric nurses regarding the need for prescriptive authority in KwaZulu‐Natal	To describe the experience of advanced psychiatric nurses regarding the need to prescribe mediation treatment in KwaZulu‐Natal	Qualitative design	Non‐probability sampling; Advanced psychiatric nurses (27)	Prescribing role of the advanced psychiatric nurse: Participants felt frustrated due to the lack of prescriptive authority, particularly as they could identify the most appropriate medication to use in such a setting. Competence: Participants felt their competencies were not fully utilised in mental health prescribing. Role recognition: Legislation should be altered to authorise prescribing for advanced psychiatric nurses, not to remove the role or importance of the psychiatrist, but rather to enhance multidisciplinary approaches and service delivery. Formulation of guidelines could serve as a directive, and to establish baselines. Furthermore, it will acknowledge their competencies.
Yang et al. (2021)	4	Patterns of mental health service use among Medicaid‐insured youths treated by nurse practitioners and physicians: A retrospective cohort study	To identify new users of psychotropic medications initiated by nurse practitioners and physicians among Medicaid‐insured youths and to assess if receiving psychosocial services prior to concurrent with medication initiation differs among youths treated by provider and specialty type	Quantitative design	A total 12 991 Medicaid‐insured youths aged 0–20 years who started psychotropic medications prescribed by nurse practitioners or physicians with primary care or psychiatric specialty during 2013–2014	Prescribing patterns: Psychiatric NPs initiated statistically significant higher levels of psychotropics in smaller metropolitan areas and in white patients (*p* < 0.01). Primary care NPs had a similar increase in metropolitan prescription. Psychiatric NPs prescribed significantly less (3.09% vs. 6.91%) psychotropics to no diagnosis patients (p value = 0.01), and statistically more (19.38% vs. 12.60%) to patients with diagnosed with depression and anxiety (p value < 0.001). No statistically significant differences for drug class prescriptions by NPs versus physicians, however, for primary care providers more antidepressants were prescribed (13.5% vs. 10.5%). Likelihood of NPs initiating prescriptions: Lower likelihood of psychiatric NPs initiating prescription for patients with no diagnosis than primary care NPs (0.42 vs. 3.76). Lower occurrences seen in those with two or three or more psychiatric diagnoses (0.64 and 0.51). Prescription to youth receiving psychosocial services: Youths who received psychosocial services prior to medication initiation were less likely to have primary care physicians (AOR = 0.15) or primary care NPs (AOR = 0.16) as their initiating prescriber than those who did not.
Chai et al. (2024)	4	Trends in incident prescriptions for behavioural health medications in USA, 2018‐2022	To assess changes in incident prescriptions dispensed for medications commonly used to treat depression, anxiety, attention‐deficit/hyperactivity disorder, and opioid use disorder, before and during COVID‐19 pandemic	Quantitative design	IQVIA National Prescription Audit; purposive sampling	Incident prescriptions: Increased from 51 500 321 pre‐COVID‐19 pandemic to 54 000 169 during the pandemic. Increase in prescription by NPs: Antidepressants (29%), benzodiazepines (7%), C‐II stimulants (57%), nonstimulant ADHD (74%), and buprenorphine (78%).

Abbreviations: ADHD, attention deficit hyperactivity disorder; AOR, odds ratio adjusted for an enrolee's age, gender, race/ethnicity, region of residence, and Medicaid eligibility; APRNs, advanced practice registered nurses; BDI‐II, beck depression inventory for patients with a primary diagnosis of depression; CGI, clinical global impression of improvement scale to assess the patient's perception of improvement in their health problem; CSQ‐8, client satisfaction questionnaire to measure satisfaction with treatment from the main care provider/organisation; MARS, medication adherence report scale to measure medication adherence; PIMs, potentially inappropriate medicines; SIMS, satisfaction with information about medicines scale to measure satisfaction with medication information; SSI, supplemental security income; SSRI, selective serotonin reuptake inhibitor; TANF, temporary assistance for needy families; TCA, tricyclic antidepressant; TGA, third generation antidepressant; WSAS, the work and social adjustment scale to assess social functioning and impairment.

^a^ Level of evidence: Level 2: Evidence from at least one well‐designed RCT (e.g., large multi‐site RCT); Level 3: Evidence from a single well‐designed controlled trial without randomisation (aka quasi‐experimental studies) OR a systematic review of a complete BOE (integrative review of higher and lower evidence) OR mixed methods intervention studies; Level 4: Evidence from well‐designed case–control or cohort studies; Level 6: Evidence from a single descriptive or qualitative study, EBP, EBQI and QI projects (Melnyk and Fineout‐Overholt 2023).

^b^ Split therapy: The treatment model in which a patient sees a clinician for psychotherapy and another clinician for medication management (Jacobs 2005).

^c^ Integrated therapy: The treatment model in which a patient sees a clinician for both medication management and psychotherapy (Jacobs 2005).

^d^ One item depression scale: Quick rating of depressed mood in patients with anxiety disorders: to measure depression in patients with anxiety and schizophrenia (Norman et al. 2010).

^e^ Median ranked score (25%, 75% interquartile ranges): median ranked score ranged from 0 to 10, where 0 = never, 5 = sometimes, 10 = always (Norman et al. 2010).

#### 3.2.1 Prescribing Patterns

The theme of prescribing patterns of nurses emerged based on 14 publications (Alexander and Schnell 2019; Chai et al. 2024; Fisher and Vaughan‐Cole 2003; Flenniken 1997; Garrison and Levin 2000; Gumber et al. 2011; Jacobs 2005; Norman et al. 2010; Poot et al. 2020; Shell 2001; Talley and Richens 2001; Wolfe et al. 2008; Yang et al. 2018; Yang et al. 2021). This theme explored the similarities and trends in prescribing practices of common classes of neuropsychiatric medication between nurses and medical practitioners (physicians or psychiatrists), the differences in prescribing were also noted. All 14 publications highlighted the growing role of nurses prescribing neuropsychiatric medications in mental health disorders (Alexander and Schnell 2019; Chai et al. 2024; Fisher and Vaughan‐Cole 2003; Flenniken 1997; Garrison and Levin 2000; Gumber et al. 2011; Jacobs 2005; Norman et al. 2010; Poot et al. 2020; Shell 2001; Talley and Richens 2001; Wolfe et al. 2008; Yang et al. 2018; Yang et al. 2021).

Flenniken (1997) and Wolfe et al. (2008) found that 76% (*n* = 32/42) of NPs (Alexander and Schnell 2019) and 65.9% (*n* = 118/179) of APNs (Flenniken 1997) prescribed neuropsychiatric medication, which were most frequently antidepressants and anxiolytics, respectively (Alexander and Schnell 2019; Flenniken 1997). During 2012 to 2014, Yang et al. (2018) reported proportional increases of neuropsychiatric medication prescriptions by psychiatric (5.9%–8.8%) and non‐psychiatric NPs (4.9%–6.3%) (Yang et al. 2018). Chai et al. (2024) expanded on this view from 2018 to 2022 in the USA, where a further increase in incident prescriptions by NPs across five drug classes was noted (Poot et al. 2020). These classes included antidepressants, benzodiazepines, Schedule II controlled substances (C‐II) stimulants, non‐stimulant attention deficit hyperactivity disorder (ADHD) drugs, and buprenorphine medications for opioid use disorder products (Poot et al. 2020). Antidepressant prescriptions by NPs increased from 6 383 463 (22%; April 2018 to March 2020) to 8 216 638 (26%; April 2020 to March 2022) (Poot et al. 2020).

It was further indicated in 4 out of 14 publications that selective serotonin reuptake inhibitors (SSRIs) were the most frequently prescribed antidepressants for depression (Fisher and Vaughan‐Cole 2003; Flenniken 1997; Gumber et al. 2011; Shell 2001). Garrison and Levin (2000) found that the majority of all prescriber groups, whether NPs (*n* = 102), physician assistants (*n* = 121), non‐psychiatric physicians (*n* = 89), or psychiatric physicians (*n* = 89), preferred fluoxetine for major depression alone, and paroxetine for concomitant anxiety and depression (Garrison and Levin 2000). Similarly, Shell (2001) observed that fluoxetine (43%) and paroxetine (39%) were the two most popularly prescribed antidepressants by NPs (Fisher and Vaughan‐Cole 2003). Yet, Talley and Richens (2001) found that the two most commonly prescribed medications were SSRIs and tricyclic antidepressants (TCAs) (Chai et al. 2024). The choice of drug was determined based on previous side effects, previous response to medication, current medical problems, and suicidality or addiction (Chai et al. 2024). This finding is consistent with a study by Gumber et al. (2011) who indicated that non‐medical prescribers' commonly prescribed antidepressants were SSRIs followed by TCAs (Gumber et al. 2011). Fisher and Vaughan‐Cole (2003) reported that psychiatrists were more likely to prescribe TCAs versus advanced practice registered nurses' (APRNs) prescriptions (19% vs. 4%) (Shell 2001). However, the overall patterns of prescriptive authority by APRNs were exercised in ways that were similar to psychiatrists (Shell 2001). Norman et al. (2010) did not find significant differences in patients' medication adherence, health status and/or side effects experienced (Jacobs 2005). Alexander and Schnell (2019) further indicated that among disadvantaged populations, there was an increased rate of prescription of antidepressants (8%) and antipsychotics (10%) by NPs as compared to counties in the USA, with adequate mental healthcare resources where NPs are authorised to independently prescribe (Norman et al. 2010).

Furthermore, Poot et al. (2020) showed that NPs prescribed lower rates of potentially inappropriate medicines (PIMs) to older adults in comparison to other prescribers in New Zealand. However, the two most frequently prescribed neuropsychiatric drug classes of PIMs by NPs were TCAs and benzodiazepines (Wolfe et al. 2008). Jacobs (2005) found that in both split and integrated therapies for the treatment of depressive disorders, there were minimal differences in the prescription frequency of SSRIs by psychiatrists (split therapy; 68% and 75%, respectively) and psychiatric nurses (integrated therapy; 75% and 73%, respectively) (Talley and Richens 2001). Similarly, Yang et al. (2021) found no major difference in a class of psychotropic medications initiated by NPs and physicians apart from a higher proportion of antidepressants prescribed (13.5% vs. 10.5%) (Yang et al. 2021).

#### 3.2.2 Perceptions and Attitudes

Nine studies reported the perceptions of nurses, patients and medical practitioners towards nurse neuropsychiatric prescribing (Gumber et al. 2011; Jacobs 2005; Norman et al. 2010; Allsop et al. 2005; Jacobs and Mkhize 2021; Jones 2005; Jones et al. 2007; Ross 2015; Snowden 2010). The broadly positive views towards nurse prescribing in mental health disorders were highlighted in eight out of the nine studies which showed evidence to support the enhanced mental healthcare, specifically pharmacotherapy (Jacobs 2005; Norman et al. 2010; Allsop et al. 2005; Jacobs and Mkhize 2021; Jones 2005; Jones et al. 2007; Ross 2015; Snowden 2010). Alexander and Schnell (2019) reported that mental healthcare in underserved areas in the USA improved since NP independent prescriptive authority was approved, which ultimately reduced the number of mental health‐related mortalities (e.g., suicides) (Norman et al. 2010). Authorisation enabled psychiatric nurses in the USA to provide not only psychotherapy, but also pharmacotherapy, which improved mental healthcare patient outcomes compared to receiving predominantly pharmacotherapy alone from psychiatrists (Norman et al. 2010). Allsop et al. (2005) and Jones et al. (2007) found that patients had a positive experience with NPs (Allsop et al. 2005; Jones et al. 2007). Allsop et al. (2005) reported that supplementary prescribing was an effective way to increase service delivery in a quicker and accessible way (Allsop et al. 2005), and that nurses believed it reduced the delay in patient access to medication and enhanced relationships with their patients in comparison to doctors (Allsop et al. 2005). Jones (2005) supported such improvements due to the large amount of time nurses spend developing and sustaining patient relationships, which could result in the nurse becoming the ‘familiar face on the ward’. (Jones 2005) Additionally, nurse prescribing allowed patients to receive a more detailed explanation of indications and possible side effects about their prescribed medication thus enhancing patient care and understanding thereof (Jones 2005). Norman et al. (2010) reported that patients' overall care satisfaction was comparable between the nurse supplementary and independent prescribers' group, with supplementary nurse prescribers satisfaction reporting being higher than independent medical prescribers (Jacobs 2005). Ross (2015) supported that patients were comfortable with nurse prescribers given their treatment discussion being better understood by patients, which improved medication adherence (Ross 2015). Other healthcare practitioners, such as psychiatrists, viewed the implementation as a benefit to patients (Jones 2005; Jones et al. 2007; Ross 2015) and considered it to be evidence‐based (Jones et al. 2007). Snowden (2010) highlighted the holistic care approach provided by nurse prescribers and found that integrating medicines management into mental health nursing improved the quality of care and patient outcomes in the UK (Snowden 2010). A single study in South Africa reported frustration among advanced psychiatric nurses in Kwa‐Zulu Natal regarding mental healthcare (Jacobs and Mkhize 2021). Advanced psychiatric nurses felt that their role was underutilised due to the inability to prescribe neuropsychiatric medication, especially during emergency situations (Jacobs and Mkhize 2021). As a result they were required to wait for a medical doctor to initiate pharmacotherapy when managing serious side effects of current neuropsychiatric pharmacotherapy or episodes of aggressive behaviour (Jacobs and Mkhize 2021). Nurse neuropsychiatric prescribing authorisation was viewed positively as a way to provide comprehensive care autonomously, ultimately enhancing their scope of practice and quality of advanced nursing practice (Jacobs and Mkhize 2021).

There were two studies across the literature which showed negative views towards the implementation (Gumber et al. 2011; Jones 2005). For example, Jones (2005) argued that psychiatrists were hesitant to accept nurse prescribing, mainly in in‐patient settings, due to a belief that the diagnosis and treatment of mental health disorders was within their sole domain given their skills and training to perform the role (Jones 2005). Similarly, nurses expressed concerns about inappropriate prescribing due to the fear of ‘doing wrong’ during prescribing (Jones 2005). Gumber et al. (2011) found that while non‐medical prescribers viewed the implementation positively, 60% believed there was inadequate training and supervision to prescribe (Gumber et al. 2011). Furthermore, conflict with psychiatrists were also reported due to the prescribing overlap among non‐medical prescribers (nurses) and psychiatrists (Gumber et al. 2011). Nurse prescribers argued that they legally prescribed at the same level of medical prescribers (psychiatrists) (Gumber et al. 2011). Coupled to this, the increase in workload and fear of litigation further emphasised the occurrence of interprofessional tension (Gumber et al. 2011).

#### 3.2.3 Auditing

A few countries have implemented nurse prescribing for mental healthcare patients, including the UK, USA and New Zealand; however, neuropsychiatric prescribing is excluded within the South African nursing Scope of Practice, thus limiting their prescribing rights (Baker and McCann 2001). According to the four articles which explored the theme of auditing (Gumber et al. 2011; Jacobs and Mkhize 2021; Ross 2015; Snowden 2010), three were based under the UK laws and regulations which have implemented the authorisation of neuropsychiatric prescribing by nurses since 2003 (Gumber et al. 2011; Ross 2015; Snowden 2010). A clear observation among the studies showed the importance of the role of auditing to ensure high standards of care, the presence of necessary legislative guidelines in place and prescriber compliance to these guidelines to safely prescribe (Gumber et al. 2011; Ross 2015; Snowden 2010). Gumber et al. (2011) and Ross (2015) both emphasised the need for the monitoring of nurse prescribing practices, promoting accountability of nurses and ensuring nurse compliance to clinical guidelines to support the management of neuropsychiatric medications (Gumber et al. 2011; Ross 2015). Furthermore, auditing procedures that are correctly implemented support compliance to clinical guidelines and ensure safe and effective prescribing decisions (Gumber et al. 2011; Jacobs and Mkhize 2021; Ross 2015; Snowden 2010). In South Africa, laws and regulations do not permit neuropsychiatric prescribing practices by nurses as no authorisation is in place (Jacobs and Mkhize 2021). As a result, Jacobs and Mkhize (2021) described the frustrations experienced by nurses created by the lack of authorisation, which they believed impacted on their nursing care (Jacobs and Mkhize 2021).

Based on the document analysis, an authorised prescriber refers to ‘a medical practitioner, dentist, veterinarian, practitioner, nurse or other person registered under the Health Professions Act, 1974’. Prescribing medication is a complex procedure whereby the prescriber requires in‐depth biological and pharmacological knowledge along with high quality communication skills (Skingsley et al. 2005) and the ability to diagnose (Gazette 1965). The Medicines and Related Substances Control Act (No 101 of 1965) states that prescriptive authority is authorised by legislation, through which the prescriber may give written directions regarding specific medication and the dosage, frequency and route of administration that should be complied to (Gazette 1965). Additionally, according to the Medicines and Related Substance Control Act (No 101 of 1965), nurses can prescribe Schedule 1 to 4 controlled substances, however there are currently no regulations in South Africa authorising nurse prescribing of neuropsychiatric medications (Schedule 5–6 controlled substances) (Gazette 1965). To authorise nurse prescription rights and extension of nurse's scope of practice, amendments are required to the Medicines and Related Substances Act, 1965 (Act 101 of 1965) and Nursing Act, 2005 (Act 33 of 2005). As a result, SAHPRA provides guidance on the process for amending Schedules to the Medicines and Related Substances Act, 1965 (Act 101 of 1965) to allow prescription rights to authorised health professionals other than medical practitioners or dentists, in accordance with the provisions of Section 22A of the Act, and states ‘SAHPRA will consider a complete and comprehensive application for an amendment to the Schedules to allow a specific authorised prescriber access to prescription rights. Once the Authority has resolved to recommend such a list, a draft set of proposed changes to the Schedules will be published to allow for comment by interested persons and institutions. Thereafter, a final set of recommended changes will be submitted to the Minister of Health for publication in the Government Gazette, in terms of section 37A of the Medicines and Related Substances Act, 1965 (Act 101 of 1965)’.

According to Sub‐section 22A(14) (b) and Sub‐section 22A(17) (a) of the Medicines and Related Substances Act, 1965 (Act 101 of 1965), nurses may prescribe a medicine or scheduled substance only if they have been authorised to do so by their professional council concerned, for which nurses would be SANC. Furthermore, the SAPC issued a document supporting the dispensing of prescriptions from nurses in the services of provincial and municipal departments of health authorised in accordance with Section 56(6) of the Nursing Act, 33 of 2005. The SAPC further indicated that nurses working in the public sector at facilities designated in terms of Section 56(6) of the Nursing Act, 33 of 2005, may prescribe medicines in accordance with the Primary Health Care Standard Treatment Guideline (STG) and Essential Medicine List (EML). The STG/EDL does not specify the prescriber level for each of the medicines, therefore the list is intended to cover medicines that can be initiated by medical practitioners only, as well as those that can be continued or initiated by nurses holding a permit issued in terms of Section 56(6) of the Nursing Act (Act 33 of 2005). Additionally, for amendment of Schedules, SAHPRA states that the health professional (nurse) is required to be a holder of a specific registration based on having completed a designated supplementary course or post‐graduate qualification. ‘This course or qualification must be accredited for this purpose by the statutory council concerned, as enabled in applicable legislation. The provider of such a course or qualification must also be accredited by the statutory council concerned, as provided for in the applicable legislation’. As such, amendments will be needed to facilitate the authorisation of relevant neuropsychiatric medication classes.

#### 3.2.4 Further Education

For nurses to prescribe neuropsychiatric medication, they are required to undergo additional training to be sufficiently knowledgeable to safely prescribe (Chai et al. 2024). Nine publications strongly emphasised such educational needs and continuous professional development (Alexander and Schnell 2019; Chai et al. 2024; Fisher and Vaughan‐Cole 2003; Flenniken 1997; Gumber et al. 2011; Yang et al. 2018; Allsop et al. 2005). Flenniken (1997), Shell (2001) and Allsop et al. (2005) identified educational gaps in the psychopharmacological knowledge of nurses who were prescribing neuropsychiatric medications, specifically antidepressants (Alexander and Schnell 2019; Fisher and Vaughan‐Cole 2003; Allsop et al. 2005). Moreover, Allsop et al. (2005) raised concerns regarding the course content for mental and learning disabilities nurses which lacked further neuropharmacological education (Allsop et al. 2005). As a result, most nurse respondents agreed to continue their education at a professional level to comfortably and safely prescribe (Alexander and Schnell 2019; Fisher and Vaughan‐Cole 2003; Gumber et al. 2011). Allsop et al. (2005) and Jacobs (2005) indicated that nurses acknowledged the importance of further education followed by preparation and training to fulfil the prescribing role successfully (Allsop et al. 2005; Jones 2005). Yang et al. (2018) highlighted the differences in prescribing practices of neuropsychiatric medication between NPs and physicians as a result of variations in their cohorts' educational background (Yang et al. 2018).

Allsop et al. (2005) and Gumber et al. (2011) showed that, with the provision of supervised training sessions and participation in opportunities for continued professional development, nurses' knowledge and skills were expanded (Gumber et al. 2011; Allsop et al. 2005) which could bridge the current educational gap. Furthermore, nurses were able to comply with necessary standards and guidelines to safely prescribe neuropsychiatric medication after receiving adequate training and mentorship to prescribe (Gumber et al. 2011). It was recommended that a well‐structured psychopharmacology curriculum be implemented focusing on drug interactions, side effects, and choice of medication to improve the safety of neuropsychiatric prescribing by nurses (Alexander and Schnell 2019; Fisher and Vaughan‐Cole 2003). Building on this view, Snowden (2010) emphasised the need to integrate a more comprehensive educational approach within the undergraduate mental health nursing curriculum by dedicating a medicines management module to enhance the pharmacological knowledge of nurse prescribers (Snowden 2010). Talley and Richens (2001) and Wolfe et al. (2008) found that APNs did not feel adequately trained and knowledgeable to safely prescribe (Chai et al. 2024; Flenniken 1997). Such inadequacies reinforce the need for enhancing continued education regarding dosing and switching strategies (Yang et al. 2018), as well as using additional resources like attending conferences to further the neuropsychiatric prescribing knowledge of nurses (Chai et al. 2024). Together, the abovementioned studies underscore the necessity of further education and training required to equip nurses with relevant knowledge and skills to prescribe neuropsychiatric medications safely and effectively.

#### 3.2.5 Barriers

The search included four publications which revealed several challenges impacting nurse prescribing of neuropsychiatric medications (Norman et al. 2010; Allsop et al. 2005; Jones 2005; Jones et al. 2010). Some of the identified barriers included interprofessional conflict (Allsop et al. 2005; Jones 2005), limited regulatory frameworks (Norman et al. 2010; Jones 2005), inadequate education and supervision for training (Allsop et al. 2005; Jones et al. 2007) and a risk of medication addiction or misuse (Norman et al. 2010). A. Jones (2005) and Allsop et al. (2005) observed that medical professionals such as psychiatrists and physicians did not fully support or accept neuropsychiatric prescribing by nurses and indicated that prescribing should remain within the psychiatrist's scope of pratice (Allsop et al. 2005; Jones 2005). Another prominent barrier found in the literature is the limited legal regulations and guidelines which govern neuropsychiatric prescribing by nurses (Norman et al. 2010; Jones 2005). Studies by Alexander and Schnell (2019) and Jones (2005) showed similarity in necessitating the expansion of laws and regulations to allow nurses to prescribe neuropsychiatric medication legally (Norman et al. 2010; Jones 2005). Furthermore, the current legislative restrictions within South Africa are a current barrier for nurses to prescribe, thus limiting their ability to address the treatment gap by providing neuropsychiatric pharmacotherapy (Jacobs and Mkhize 2021). Alexander and Schnell (2019) emphasised that by making regulatory changes to authorise nurses to use their full prescribing potential, the shortage of mental healthcare provision in underserved populations could be mitigated (Norman et al. 2010).

As discussed in section 4.2.4, nurses require additional supervised training and continued education to safely prescribe neuropsychiatric medication. However, further neuropharmacological training is mostly completed through the support of peer group supervision and training from experienced medical practitoners (Allsop et al. 2005). Yet, in contrast, Jones et al. (2007) identified the lack of sufficient supervision and mentorship to enable nurses to gain additional prescribing knowledge and experience (Jones et al. 2007). Jones et al. (2007) observed that nurses felt isolated in their role as a prescriber due to the limited supervision of senior prescribers available to provide mentorship to nurse prescribers (Jones et al. 2007), which Allsop et al. (2005) argue could be mitigated through integrated support in the forms of senior mentorship, continued education and interprofessional collaboration (Allsop et al. 2005). Lastly, Alexander and Schnell (2019) highlighted the risk of patients developing misuse due to the broader availability of neuropsychiatric medication (Norman et al. 2010), which could then negatively impact the implementation of nurse prescribing in mental health settings.

#### 3.2.6 Benefits

**FIGURE 2:**
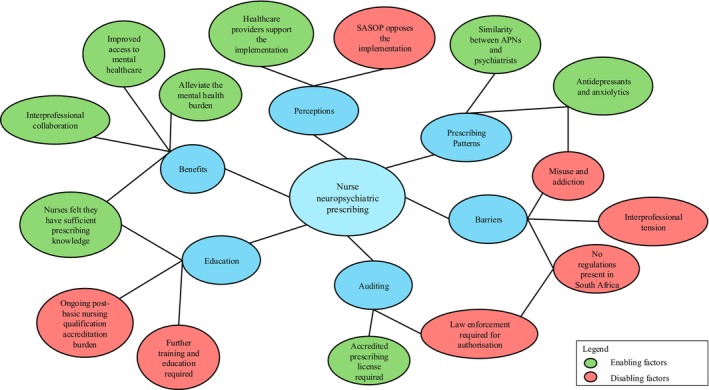
Integrated mind map illustrating the key themes that emerged from the scoping review.

The benefits of neuropsychiatric prescribing by nurses were highlighted among six studies which showed a continuous trend in increasing access to mental healthcare for patients while improving patient outcomes (Jacobs 2005; Norman et al. 2010; Yang et al. 2018; Allsop et al. 2005; Jones 2005; Ross 2015). Ross (2015) and Yang et al. (2018) highlighted the patient‐centred care provided by nurses (Yang et al. 2018; Ross 2015). Mental health nurses provide a holistic treatment approach combined with their prescribing practices (Ross 2015). As a result, there were improved patient compliance and increased medication reconciliation since nurse prescribers minimised the prescriptions of medications that patients no longer required (Ross 2015). Jones (2005) mentioned that nurses, if sufficiently trained to be nurse prescribers, could increase access to medication for patient care and resolution of patient needs (Jones 2005). Additionally, A. Jones (2005) and Allsop et al. (2005) reported that the implementation of nurse prescribing in mental healthcare settings provided an opportunity for immediate decision‐making with regard to treatment and reduced unnecessary referrals to psychiatrists (Allsop et al. 2005; Jones 2005). Nurse prescribing in mental healthcare settings further bridged the gap in accessing pharmacotherapy, as Alexander and Schnell (2019) found that nurse prescribing in underserved areas increased patient access to pharmacotherapy which showed improvement in the population's mental health outcomes (Norman et al. 2010). Similarly, Yang et al. (2018) showed that nurse prescribers play a critical role in mental healthcare provision to rural populations where access to a physician or psychiatrist is limited (Yang et al. 2018). As a result, this increases the nursing role in neuropsychiatric prescribing as compared to physicians, indicating the expansion of mental health services in these areas (Yang et al. 2018). Additionally, there were reductions in the prevalence of mental health–related deaths (suicides) in populations underserved by psychiatrists which showed difficulty in accessing physician‐provided care (Norman et al. 2010). The study also suggested that in 2014 alone, 1596 mental health‐related deaths were averted in underserved counties within the USA by authorising NPs to prescribe independently (Norman et al. 2010). Lastly, the findings by Norman et al. (2010) revealed that nurse supplementary prescribing had no significant clinical differences to independent medical prescribing, whereby similar medication adherence, health status, side effects, and satisfaction with the overall care of patients were observed (Jacobs 2005). Furthermore, the implementation of nurse prescribing in mental healthcare settings was also more cost efficient for patients as there were no significant differences in the costs of the two types of prescribing (nurse supplementary prescribers vs. independent medical prescribers) (Jacobs 2005). This suggests that mental health nurse supplementary prescribers can deliver similar health benefits to patients as consultant psychiatrists without any significant difference in patients' service utilisation costs (Jacobs 2005) (Figure 2).

## 4 Discussion

The scoping review explored the current landscape of neuropsychiatric prescribing by nurses globally. Based on the literature, the context is overtly from a first‐world country perspective given most publications originate from the UK and USA due to their current legislative approvals. The findings explored the existing impact and challenges because of the authorisation, and further highlighted the trends in practice, perceptions and views towards the implementation, education and legal policies. Furthermore, the findings show nurse prescribers to be highly beneficial in enhancing the availability of neuropsychiatric pharmacotherapy, but there are significant challenges which arise from the implementation, such as a lack of sufficient knowledge and education of nurse prescribers and limited regulatory frameworks in place that will need to be addressed.

The complexity of diagnosing a mental health disorder coupled with choosing the appropriate neuropsychiatric treatment can pose a challenge since the patient requires an individualised treatment plan to target the diverse presentation of symptoms (Kupfer 2005). Neuropsychiatric pharmacotherapy is determined based on the level of symptom severity, current functional impairment of the patient, the duration of the depression, the presence of psychotic symptoms, level of suicidality, and previous illness and treatment history (Kupfer 2005). Mental health disorders such as depressive and anxiety disorders may require neuropsychiatric pharmacotherapy which then involves medications with complex mechanisms of action. There are currently between 10 and 20 various antidepressants available, depending on the country (Schulz and Macher 2002). Antidepressants can be classified as first‐, second‐ or third‐generation antidepressants (Schulz and Macher 2002). TCAs and monoamine oxidase inhibitors are first‐generation antidepressants, and SSRIs are second‐generation antidepressants (Schulz and Macher 2002). Third‐generation antidepressants include newer molecules like nefazodone, milnacipran, mirtazapine, and reboxetine (Schulz and Macher 2002). The three generations of antidepressants mentioned above have the same level of clinical efficacy in the treatment of major depression (Schulz and Macher 2002). Yet, compounds from the second‐ and third‐generations do not elicit life‐threatening side effects in overdose, and present a more favourable display of side effects at the normal therapeutic doses (Schulz and Macher 2002). However, the complexity lies in the fact that there is no demonstration that any given class of antidepressant is more efficacious than another when treating different categories of depression (Schulz and Macher 2002). The efficacy of the drug as well as the potential side effect profile and the patient's specific condition need to be considered. Therefore, highlighting the complexity of decision‐making when prescribing the most appropriate choice of drug.

Based on results of prescribing patterns, similarities in prescribing were evident among nurses and physicians. The frequently prescribed neuropsychiatric medications were found to be antidepressants such as SSRIs and TCAs, and anxiolytics such as benzodiazepines for treating depressive and/or anxiety disorders. As indicated, a difference in choice of drug does not provide a different or lower level of therapeutic effect; therefore, it is clear that the choice in drug/drug class may differ among prescribers. However, the necessary guidelines are followed, and prescribers receive adequate training to ensure that the patient's safety is prioritised.

As highlighted, there is potential for nurse prescribing to improve patient access to mental healthcare, particularly pharmacotherapy. The NPs are often the primary mental healthcare providers within rural or underserved areas (Balestra 2019), therefore, by implementing nurse prescribing in primary mental healthcare settings, the service gap created due to insufficient psychiatrists or physicians can be bridged. From the results, it is evident that nurse prescribing could reduce unnecessary psychiatric referrals as nurses could then issue the prescription. Similarly, prescribing pathways in the UK and New Zealand were compared and highlighted that nurse prescribing enables a person‐centred approach to mental healthcare, thus reducing the burden on overextended medical professionals and leading to quicker treatment access. The results about the benefits indicate that the implementation can be a solution to improve current healthcare challenges, specifically in resource‐limited areas. This could ultimately improve access to pharmacotherapy, increase service delivery, and improve patients' mental health outcomes. However, while the benefits of increased access to neuropsychiatric pharmacotherapy are well supported, the barriers against the implementation should also be considered. It can be argued that implementing neuropsychiatric prescribing by nurses could lead to an overburden of additional responsibilities which could be detrimental in fulfilling their role successfully. Furthermore, by impulsively increasing the accessibility of neuropsychiatric pharmacotherapy, which is known to be highly controlled scheduled substances (Schedule 5–6), this could create a risk of patients misusing these medications, ultimately developing an addiction. Therefore, creating a paradoxical outcome.

The literature suggests that while the perception of neuropsychiatric prescribing by nurses is largely positive, interprofessional tension and concerns about training and supervision require investigation and intervention. Notably, some healthcare professionals, such as physicians, psychiatrists, and even nurses, did not fully support the integration of neuropsychiatric prescribing by nurses. This could be as a result of concern about nurses' competence in the successful management of complex diagnoses, especially when prescribing neuropsychiatric medications with serious side effect profiles (Jones 2008). The South African Society of Psychiatrists (SASOP) opposed the possible implementation of Objective 1.7 of the National Strategic Plan for HIV, TB, and STIs 2023–2028 (van den Berg 2023). The SASOP indicated that the plan did not take into consideration the state's currently overwhelmed psychiatric services, the shortage of psychiatric nurses to prescribe and dispense these medications, and the adverse effects of prescribing antidepressants and anxiolytics without proper psychiatric assessment and follow‐up. (van den Berg 2023) Moreover, the polypharmacy of HIV and mental health disorder medications raises concerns of possible drug–drug interactions and inadequate mental health disorder diagnosis (van den Berg 2023). The UK has shown to extend nurses' prescribing privileges in mental health disorders; however, concerns continue to persist regarding nurses' scope of practice and the adequacy of sufficient training and education (Heming Hemingway and Ely 2009). The need for clear training and education on medicine management, which are recommended to overcome these barriers, has been confirmed in previous studies. Overall, while the barriers mentioned act as a limitation to the implementation of nurse prescribing of neuropsychiatric medications, when addressed, they could improve mental healthcare access and patient outcomes.

Continued education in pharmacology and clinical decision‐making was emphasised to ensure nurse prescribers are prepared to manage the complexities of prescribing neuropsychiatric medications (Jones 2008). A lack of sufficient training can increase the risk of errors, particularly in areas such as managing drug interactions or prescribing in patients with comorbid conditions (Balestra 2019). Since most medication errors occur at the prescribing stage (Bates and Slight 2014), more guidance and education on prescription writing and pharmacology may help to mitigate some of these errors (Procyshyn et al. 2010). Examples of prescription writing errors include: (i) illegibility; (ii) ambiguous prescribing; (iii) writing an incomplete prescription; (iv) omission of the prescriber's signature; (v) major misspelling of a drug name; and (vi) prescribing without a strength for a drug (Procyshyn et al. 2010). As a result, legal risks associated with prescribing, particularly if nurses are inadequately trained or working without the support of clear clinical guidelines followed by the error causing harm to the patient, may lead to lawsuits and possibly discipline or criminal prosecution (Merry 2009). Since many neuropsychiatric medications have the potential to cause significant morbidity and mortality if used incorrectly (Nair and Srivastava 2012), the legal response will be proportionate to the actual consequences of the error, rather than to potential consequences or the moral culpability involved (Merry 2009). A thorough legislative framework and monitoring system to record nurse prescribing patterns and ensure compliance to safety protocols will be needed (Murray 2007). The role of auditing was seen as a challenge which forms an important part in shaping nurse prescribing practices. By implementing well‐defined regulatory frameworks, areas whereby nurse prescribers require additional support or training could be identified, and in doing so, the risk of medication errors may be reduced (especially in patients with a complex mental disorder who requires polypharmacy). For example, South Africa permits certain healthcare professionals, like nurses, to prescribe specific schedules of controlled medication, permitting they acquire a Section 22A (15) permit. One of the requirements of nurses to become a Section 22A (15) permit holder is to have completed an additional (post‐basic) nursing qualification. All post‐basic nursing qualifications require alignment with Higher Education Qualifications Sub‐Framework (HEQSF) and accreditation from the SANC, Council of Higher Education (CHE) and the South African Qualifications Authority (SAQA) (Crowley and Daniels 2023). However, there are challenges with the external accreditation processes of these post‐basic qualifications since several nursing education institutions' postgraduate diploma nursing programmes are not fully accredited by SANC, CHE and the SAQA (Crowley and Daniels 2023). As indicated in the literature, nurses require additional education to safely prescribe neuropsychiatric medication, and as a result, this additional qualification could add to the current Mental Health Nursing postgraduate diploma qualification accreditation burden which could then lengthen the process of authorising nurses to prescribe neuropsychiatric medication (Crowley and Daniels 2023).

Furthermore, due to the high risk of dependence, misuse, and adverse events, prescribing of neuropsychiatric medication requires continuous drug monitoring and regular clinical assessments including follow‐ups (Norman et al. 2010). However, due to the rising number of people living with a mental health disorder, coupled with the shortage of mental health care providers (Cuijpers et al. 2021), the healthcare system becomes overburdened which could impact the follow‐up required in patients receiving neuropsychiatric pharmacotherapy. A main concern is the risk of misuse and addiction that a patient can develop with neuropsychiatric medications (Norman et al. 2010). Continuous monitoring and follow‐up care are required to avoid these risks, thus ensuring patient safety and effective treatment outcomes. However, the current healthcare environment poses some challenges. This includes resource constraints, such as increased workloads of healthcare providers and insufficient staffing, more so in mental health nursing (López‐López et al. 2019). This can limit the ability of nurses to provide the necessary follow‐up care due to the high rates of patients. Nurses are commonly affected by burnout due to the increased stressors thus further impacting the inability to provide follow‐up care (López‐López et al. 2019) which then contributes to workforce turnover and impacts the stability of care in mental health settings (Sumner and Townsend‐Rocchiccioli 2003). Therefore, to address these challenges, actionable strategies are required.

After the declaration of the COVID‐19 pandemic, people with pre‐existing mental health and substance use disorders were at an increased risk of infection with COVID‐19, increased inaccessibility to testing and treatment, and increased risk of negative physical and psychological effects stemming from the pandemic (Cullen et al. 2020). Due to limitations on in‐person contact resulting from the risk of viral transmission, telemedicine was implemented and has proven effective in addressing mental health needs during the COVID‐19 pandemic (Mendonça et al. 2022). Similarly, the integration of digital technologies, such as telemedicine in follow‐up mental healthcare and remote patient monitoring, can enhance patient assessment during their use of neuropsychiatric medications and ultimately reduce the burnout rate of nurses. Given the relatively capped supply of traditional mental health treatments and the need to meet the ever‐increasing demand, digital mental health interventions are likely to be an unavoidable part of any solution to treatment access issues (Aboujaoude et al. 2020).

## 5 Conclusions and Recommendations

Nurse prescribing increases the therapeutic options available to patients, and it is this enhanced therapeutic relationship that most likely supports the benefits of nurse prescribing suggested in current literature. However, as of yet it is unclear whether the South African healthcare system will be able to support such an initiative, and what the potential beneficial and/or detrimental consequences of it may be depending on implementation success. Although there is support for the approval of nurses to prescribe neuropsychiatric medication from the obtained literature within their countries, contextual research will be necessary to ascertain whether South African stakeholders will support such an authorisation and whether it will be feasible. Investigation of potential professional overlap of responsibilities and perceptional biases, as well as transformation of educational platforms will be needed should such a recommendation come to pass. Furthermore, legislative changes will need to occur to authorise such a practice. By addressing these considerations, nurse prescribing has the potential to transform mental healthcare delivery and improve outcomes of people living with a mental disorder.

## 6 Limitations

There was minimal agreement between reviewers during the full‐text screening process, as indicated by a low Cohen‐kappa statistic value. This discrepancy was due to a differential view between reviewers regarding what was considered primary research. The reviewers mitigated this through thorough consensus discussion to finally decide on included publications. Furthermore, little information was available beyond the USA and UK and was not recent publications. As such, current trends and perceptions may not be reflected.

## Author Contributions

**M. Naidoo:** writing – original draft, writing – review and editing, visualisation, investigation, formal analysis, data curation. **C. J. Filmalter:** writing – review and editing, supervision. **W. Cordier:** writing – review and editing, supervision, conceptualisation, methodology, investigation, formal analysis, data curation.

## Ethics Statement

The following study obtained ethics approval from the [Research Ethics Committee of the Faculty of Health Sciences (REC 181/2024)]. The conduction of the two‐process screening method was blinded until the reviewers completed screening to allow for an unbiased decision when selecting citations. During the conduction of the document analysis, direct quotations obtained from the documents were discussed contextually using coding to establish themes. Verbatim quotation of the acts and guidelines was done to ensure transparency and reliability, where the multiple reviewer approach allows for inter‐rater discussion of the information.

## Conflicts of Interest

The authors declare no conflicts of interest.

## Data Availability

The data that support the findings of this study are available from the corresponding author upon reasonable request.
